# The Influence of Culture, Social, and Religious Support on Well-Being in Breast Cancer Survivorship

**DOI:** 10.7759/cureus.14158

**Published:** 2021-03-28

**Authors:** Nathaniel J Flores, Mary J Mathew, Leah S Fortson, Alexis D Abernethy, Kimlin T Ashing

**Affiliations:** 1 School of Psychology & Marriage and Family Therapy, Fuller Theological Seminary, Pasadena, USA; 2 Department of Clinical Psychology, Azusa Pacific University, Azusa, USA; 3 Department of Population Sciences, City of Hope, Duarte, USA

**Keywords:** breast cancer survivors, well-being, health disparities, social support, ethnic salience, religious support

## Abstract

Objective

Latina and African American breast cancer survivors (BCS) are affected by health disparities that have negatively impacted their health outcomes and quality of life more than other BCS. Examining the relationships among social support, culture, and well-being in underserved groups may help clarify critical factors that influence health disparities in cancer survivors.

Methodology

Ethnic salience (impact of ethnicity on identity), religious support, social support, and well-being were examined in African American and Latina breast cancer survivors using archival data. Participants included 320 breast cancer survivors (28% African American and 72% Latina) ranging from 26-89 years old and one to five years post breast cancer diagnosis.

Results

Ethnic salience was positively associated with well-being (p < .001). African American breast cancer survivors endorsed greater well-being, social support, religious support, and ethnic salience than Latinas (ps < .05). Religious support was associated with well-being even after controlling for the effects of general social support [*ΔR*^2^ = .02, *p* = .005; *F*(5, 298) = 23.67].

Conclusion

Ethnic salience and religious support are important factors in understanding health disparities and should inform survivorship care plans for underserved populations.

## Introduction

Breast cancer is the most frequently diagnosed and the second leading cause of death among women [1]. African American breast cancer survivors (BCS) have slightly lower incidence, 126.5 versus 130.1 per 100,000, and higher mortality rates, 28.9 versus 20.6 per 100,000, as compared to Caucasian women; Latinas have a 93.0 per 100,000 incidence rate and 14.3 per 100,000 mortality rate [1-2].

Socioeconomic status (SES) has been associated with greater health disparities and higher death rates for ethnic minorities [3]. Additional cultural factors that exacerbate health disparities include a lack of knowledge about breast cancer, medical care issues, cost, language barriers, and cultural factors related to illness beliefs [4]. Although 70% of women over 40 report having a recent mammogram, screening varies by ethnicity, education, access to care, and acculturation [5].

Cancer survivorship

Concerns for breast cancer survivors encompass a lack of language concordance, literacy issues, and cultural preferences regarding interpersonal communication [6]. Different needs arise for breast cancer survivors across the survivorship continuum: external and interpersonal factors related to economic issues, relational challenges, and concerns with sexuality/body image [7]. Breast cancer survivors’ concerns may vary among different ethnic groups during and after the cancer treatment process.

African American BCS report lower rates of adjuvant therapy than Caucasian BCS as well as behavioral and cultural differences that impact survival rates [3,8]. Latina BCS, who were less acculturated, had difficulty understanding written materials about treatment and survivorship-related information and had more unmet information and care support needs than Whites and African American women [9]. Breast cancer survivorship care plans (SCP) that have addressed ethnic and linguistic considerations have been associated with a higher quality of care and have been modified to include better communication among medical staff and patients [9-10].

Several models have been helpful in understanding survivorship. This study builds upon prior work to include the culturally contextualized model of Health-Related Quality of Life (HRQoL) that addresses the sociocultural context such as the physical, functional, psychological, social, spiritual, and sexual well-being of individuals [10]. The Contextual Model of the HRQoL [10] includes individual and systemic dimensions: (1) socio-ecological context, (2) cultural context, (3) demographic context, (4) healthcare system context, (5) cancer-related medical factors, (6) general health and comorbidity, (7) health practices and utilization, and (8) psychological well-being (p. 298-299).

Social support, religious support, and ethnic salience may be understood as the socio-ecological and cultural components of this model that may represent both latent and explicit determinants of health disparities [4].

Social support

Social support was initially defined in broad but non-contextual ways. Early studies examined qualitative (e.g., structure, content, function of social network), quantitative support (e.g., anchorage, reachability, density, range) [11], and types of support available such as emotional support and additional assistance from friends [12]. More recently, the discussion of social support has included esteem support, instrumental support, informational support, and social companionship [13]. Social support has become a multidimensional construct that can be assessed through both structural and perceived social support [14].

Some of the major changes in studying social support included a shift from quantitative to qualitative studies, to perceived and received social support, and toward more contextual considerations such as culture [15-16]. These contextual approaches have been evident in cancer studies as well. In past research, emotional support was especially helpful depending on the provider of the support and differed depending upon gender and race [17]. These findings highlight the importance of considering an individual’s cultural contexts in understanding social support.

Longitudinal analyses indicated that social support was an important predictor of HRQoL in women diagnosed with breast cancer [18]. Alferi and colleagues found that emotional support was higher than informational support in low SES Hispanic breast cancer patients during all tests pre- and post-surgery [19]. Acknowledging the importance of social networks and increasing sources of social support for Caucasian, Latino/Hispanic, and African American women diagnosed with breast cancer were critical factors in both increasing prevention screenings and well-being pre- and post-treatment [20].

Religious support

Cancer patients who have religious beliefs may experience an enhanced sense of social support from a community with whom they share those beliefs [21]. Religious support is a cultural component within the HRQoL model that functions as an extension of the larger socio-ecological variables that influence well-being. Religious support includes formal support from church leaders, the congregation, and direct support from God [22]. Religious support is a functional measure of an individual’s perceived assistance from both the divine and their religious community [23].

Religious support may be understood as a cultural component of perceived social support that may have a positive or negative impact on well-being [24-25]. Higher levels of quality of life have been associated with religious support for advanced cancer patients [25]. Assistance included the support of spiritual needs provided by staff within the medical system (e.g., doctors, nurses, and chaplains) and the religious community. Additionally, qualitative research has examined the importance of religiousness and spirituality in coping with cancer, citing spiritual resources that included a relationship with God, religious coping activities (e.g., prayer), social support, and meaning [4]. Both African American and Latina BCS reported religion and spirituality as important aspects of their daily life, and these factors were associated with higher social and functional well-being [7,26-27].

The present study

This study is a part of a larger intervention breast cancer survivors study coordinated by the corresponding author and funded by the Department of Defense. This current study was designed to examine the effects of social support, religious support, and ethnic salience (i.e., the degree of identification with one’s culture) on well-being among Latinas and African American women diagnosed with breast cancer.

The following hypotheses were examined:

Individuals who report higher ethnic salience would have increased social support.

Individuals who endorse higher ethnic salience would have higher well-being.

African Americans would endorse higher scores on well-being, social support, religious support, and ethnic salience as compared to Latinas.

For Latinas, English speakers would report greater well-being, greater social support, and higher ethnic salience than Spanish speakers.

Religious support would be associated with well-being even after controlling for social support.

The relationship between ethnic salience and social support would vary depending on the level of religious support.

The relationship between ethnic salience and well-being would vary depending on the level of religious support.

## Materials and methods

Participants

Study participants were recruited for a larger educational intervention study examining breast cancer survivors. This study focused on the pre-assessment phase. Study participants (N= 320) were recruited from state and local hospital cancer registries in Southern California and gave informed consent. The participants were breast cancer survivors (28% African American, 73% Latina) who ranged in age from 26 to 89 (M = 54.33, SD = 11.85). Participants were selected based on their ethnicity, time of diagnosis (i.e., between 1 and 5 years), stage of breast cancer (stages I-III), the absence of comorbid medical or psychiatric conditions, and their ability to read and speak either English and/or Spanish.

Procedure

In the original study, potential participants received a recruitment letter, informed consent forms, and a self-addressed, postage-paid envelope by mail. Verbal consent was obtained prior to the screening. Interested candidates were given a brief telephone screening to determine their eligibility. Eligible participants were then asked to mail the signed consent form. After the signed consent forms were returned, participants were mailed a packet containing the survey questionnaire. Surveys were returned in a postage-paid return envelope.

Measures

Demographic

Demographic questions were designed to assess the age, ethnicity, income, education, employment status, country of origin, insurance coverage, language, and whether individuals applied for disability or social security benefits.

Social Support

The Medical Outcomes Study (MOS) is a 19-item five-point subscale. Within the MOS, there are four subscales assessing the individual’s emotional/informational support, tangible support, affectionate support, and positive social interaction that combine to form a total score. Participants were asked to respond to statements using a five-point response scale ranging from (1) none of the time to (5) all of the time. Cronbach’s alpha was 0.91.

Religious Support

The religious support measure was one item developed by Dr. Ashing in assessing the support received from the church and the spiritual or religious community to help breast cancer survivors deal with difficult situations. Participants were asked to respond to the statement using a five-point response scale ranging from (1) not at all to (5) very much. Cronbach’s alpha was 0.88.

Ethnic Salience

The Ethnic Identity Scale is a five-item measure developed by Dr. Ashing to assess the level of impact or identification with ethnicity. Within the Ethnic Identity Scale, there are items assessing the impact of ethnicity on identity, a motivator to become the best that I can be, concern about the well-being or status of my group in this society, and participation in the native practices with those of other cultures. The scale was summed to capture cultural resistance, cultural incorporation, and cultural shift in the ethnic salience scale. Cronbach’s alpha ranged from .84 -.91 for this scale.

Functional Assessment of Cancer Therapy - Breast (FACT-B)

The Functional Assessment of Cancer Therapy - Breast (FACT-B) is a 37-item four-point subscale derived from the Functional Assessment of Chronic Illness Therapy (FACIT) scales. Within the FACT-B, there are five subscales assessing an individual’s physical well-being, social/family well-being, emotional well-being, functional well-being, and additional concerns that combine to form a total score. Participants were asked to respond to statements using a four-point response scale ranging from (1) not at all to (4) very much. Cronbach’s alpha was .92 for this scale.

Data analysis

Statistical analyses were performed using the Statistical Package for the Social Sciences (SPSS) version 24.0. Descriptive statistics were conducted for all study variables. A multivariate analysis of variance (MANOVA) was conducted on the dependent variables (e.g., age, ethnicity, race, location born, born in the US, education, occupation, income, stage at diagnosis, health insurance, and whether the patients had applied for or received disability or Social Security benefits since having breast cancer) on ethnic salience, religious support, social support, and well-being to determine whether demographics variables needed to be controlled in subsequent analyses. Income was controlled for when social support or well-being were outcomes variables and disability/social security benefits also needed to be controlled for when well-being was the outcome variable. Frequency statistics were conducted on all test variables to determine whether scores were normally distributed. Scores on social support (-.63) and well-being (-.55) were skewed. This was consistent with the literature that suggests breast cancer survivors have a relatively good overall quality of life (QOL) [28].

## Results

A multivariate analysis of variance (MANOVA) was conducted among demographic variables (e.g., age, ethnicity, race, location born, born in the US, education, occupation, income, stage at diagnosis, health insurance, and whether the patients had applied for or received disability or Social Security benefits since having breast cancer) on well-being, perceived cognitive decline and factors affecting perceived cognitive decline. See Tables 1-2.

**Table 1:** Demographic characteristics of the sample Note: Sample size = 320; n indicates frequency, % indicates percentage, M indicates mean, and SD indicates standard deviation

Variable	n	%	M	SD
Ethnicity			1.73	.45
African American	88	27.5		
Latina	232	72.5		
Language			1.43	.50
English-Speaking Latinas	95	40.9		
Spanish-Speaking Latinas	137	59.1		
Stage at Diagnosis			1.68	.81
Stage 0	17	5.3		
Stage I	119	37.2		
Stage II	127	39.7		
Stage III	52	16.3		
Racial/Ethnic Background			2.49	1.02
Black-African	90	28.1		
White	2	.6		
Hispanic/Latina	222	69.4		
Bi-/Multiracial	6	1.9		
Education			3.69	2.08
Grade School	69	21.6		
Some High School	44	13.8		
High School Grad	45	14.1		
Vocational	28	8/8		
Some College	64	20.0		
College Grad	34	10.6		
Masters	27	8.4		
Doctoral Degree	7	2.2		
Income			3.32	2.20
Under 15k	94	29.6		
15-25k	58	18.2		
25-35k	37	11.6		
35-45k	36	11.3		
45-60k	20	6.3		
60-75k	21	6.6		
Over 75k	52	16.4		
Disability/Social Security (Applied, Receiving)			2.53	.90
Yes, Receiving	57	17.8		
Yes, Not Receiving	67	20.9		
No, Never	162	50.6		
No, Already On	31	9.7		

**Table 2:** Descriptive statistics and correlations for study variables Note: Sample size = 320; n indicates frequency, M indicates mean, and SD indicates standard deviation, ** = significant at the 0.01 level (2-tailed), Ethnic Salience and Religious Support are IVs, and Social Support and Well-Being are DVs

Variable	n	M	SD	1	2	3	4
Ethnic Salience	320	10.53	3.54	-			
Religious Support	316	2.96	1.53	0.22**	-		
Social Support	311	71.91	17.69	0.10	0.24**	-	
Well-being	320	79.18	17.22	0.08	0.21**	0.46**	-

The first hypothesis that ethnic salience would be positively associated with social support was not supported (r = .09, p = .88). The second hypothesis that ethnic salience would be positively associated with well-being was supported as breast cancer survivors who endorsed higher ethnic salience endorsed a greater impact on well-being (r = .09, p < .001).

A MANOVA was conducted to test the hypotheses that African Americans would endorse more well-being, greater social support, greater religious support, and higher ethnic salience as compared to Latinas. These hypotheses were supported. There was a significant group difference in well-being by ethnicity, F(1, 304) = 10.40, p < .001. The mean score for African Americans survivors (M = 83.80, SD = 16.24) reflected more well-being than Latina survivors (M = 77.86, SD = 17.55). There was a significant group difference on social support by ethnicity, F(1, 304) = 5.89, p < .001. The mean score for African Americans survivors (M = 75.61, SD = 14.98) was significantly higher social support than Latina survivors (M = 70.75, SD = 18.32). There was a significant group difference on religious support by ethnicity, F(1, 304) = 4.83, p =.001. The mean score for African Americans survivors (M = 3.27, SD = 1.66) was significantly higher than religious support for Latina survivors (M = 2.83, SD = 1.45). There was a significant group difference on ethnic salience by ethnicity, F(1, 304) = 14.70, p < .001. The mean score for African Americans survivors (M = 12.51, SD = 2.84) reflected more ethnic salience than Latina survivors (M = 9.87, SD = 3.49).

A MANOVA was conducted to test the hypotheses that English-speaking Latinas would endorse greater social support, greater well-being, higher ethnic salience as compared to Spanish-speaking Latina breast cancer survivors. These hypotheses were not supported as the groups did not differ on well-being, F(1, 220) = .01, p = .94, ethnic salience, F(1, 220) = .02, p = .89, or social support, F(1, 220) = .14, p = .71.

A hierarchical regression analysis was conducted to determine whether religious support was associated with well-being after controlling for social support. This hypothesis was supported, (β = .14, p < .05), as religious support was positively associated with well-being even after controlling for the contributions of general social support, (ΔR2 = .02, p = .005; F(5, 298) = 23.67, p < .001).

A hierarchical regression analysis was conducted to determine whether religious support moderated the relationship between ethnic salience and social support (DV). There was no main effect of ethnic salience on social support (β = .04, p = .898). There was a significant main effect of religious support on social support (β = 2.51, p < .001). There was no significant interaction between ethnic salience and religious support on social support (β = .04, p = .831).

A hierarchical regression analysis was conducted to determine whether religious support moderated the relationship between ethnic salience and well-being (DV). There was no main effect of ethnic salience on well-being (β = -.16, p = .583). There was a significant main effect of religious support on well-being (β = 2.50, p < .001). There was no significant interaction between ethnic salience and religious support on well-being (β = .07, p = .680).

## Discussion

Past research has highlighted the effects of socio-economic and cultural factors on well-being [3-4,10-11]. This study examined the effects of social support, religious support, and ethnicity on the well-being of African American and Latina breast cancer survivors using the culturally contextualized HRQoL model. These results support the importance of cultural differences and illumine the role of cultural factors in cancer survivorship. A cultural lens clarifies the cultural dimensions of health and spirituality constructs and creates a clearer framework for understanding health disparities in cancer survivorship [29].

These findings underscore the importance of religious support and ethnic differences on well-being even after controlling for social support. The results from this study indicate that religious support is an important consideration beyond the effects of social support. African Americans reported more religious support than Latina BCS. In a chronic illness, such as cancer, the social and religious support within underrepresented ethnic groups may differ. Understanding the role of religious support may identify an important unique resource for certain breast cancer survivors that is provided by individuals who share their beliefs [22].

Consistent with past research, social support was associated with well-being. Previous studies have highlighted social support as a predictor of HRQoL [18]. As hypothesized, African American BC survivors endorsed more social support and well-being than Latina breast cancer survivors. These findings support the notion that there may be a difference among ethnic groups on the effect of explicit and implicit social support [18]. For example, studies on culture and social support indicate that compared to European Americans, Asians and Asian Americans are less willing to seek explicit social support for dealing with their stressful events and benefit less from social support [18]. The differences in culture and social support for African Americans and Latinos may explain some of the health disparities in cancer survivorship. The present study built on Leung and colleagues’ finding that social support was a predictor in HRQoL and included both religious support and ethnicity as factors influencing well-being [18]. Although cross-sectional analyses were conducted in the current study, these findings point to the importance of examining the role of ethnic differences and social support in well-being over time.

Ethnic salience examines the level of impact or identification with ethnicity within the socioecological context. Findings suggest that ethnic salience was associated with well-being. As hypothesized, African American BCS endorsed more well-being and greater ethnic salience than Latina BCS highlighting group differences. While factors such as acculturation levels and diversity within this particular group of African and Latina breast cancer survivors may help explain these differences, the most important finding is that ethnic salience is associated with well-being and may be an important cultural factor in HRQoL. This highlights the importance of supporting an individual’s ethnic identification in the treatment process. Utilizing culturally tailored interventions that promote well-being, for example, offering interventions that address patients’ salient cultural values, may reduce health disparities. The hypothesis that ethnic salience would be associated with social support was not supported. A more fully developed measure of ethnic salience might help to clarify this relationship.

Language preference was not related to well-being, ethnic salience, or social support for Latina breast cancer survivors. This is an unexpected finding and is contrary to research that has demonstrated the importance of language in predicting well-being among Latina breast cancer survivors [30]. This may reflect the inclusive nature of how language preference was assessed in this study.

Some limitations of this study include the limited measurement of key constructs such as religious support; a one-item measure was used rather than a multidimensional measure with greater psychometric support. The construct of religious support may include support differences in obtaining assistance from God, one’s congregation, and religious leadership [22]. More research is needed to address the interrelationships among different areas of support endorsed by African American and Latina breast cancer survivors. The inclusion of God support within religious support would both broaden the understanding of the effects of religious support on HRQoL for breast cancer survivors. Given the association of spirituality and religious support with quality of life [25], including a religious support scale to assess the socioecological and cultural components of the Contextual Model of HRQoL, will be important in future research.

As noted earlier, perceived social support and provided support [14], as well as structural social support and perceived social support [15], were defined as important in understanding social support, thus, future studies should explore the nuances of perceived versus received support among these ethnic groups, which may help professionals working with these populations in defining how to incorporate culturally relevant aspects of social support into the survivorship care plan.

## Conclusions

This study of multiethnic and multilingual population that has experienced cancer health disparities may inform the development of interventions that are culturally sensitive that address ethnic salience and religious support, in particular. Religious support is an important factor beyond the effects of social support in determining the HRQoL of Latina and African American BCS. Greater ethnic salience was related with greater HRQoL among Latina and African American BCS. The study also offers support for the Contextual Model of the HRQoL as a valuable framework to explore the interaction of cultural factors within the contextual dimensions of breast cancer survivorship, which ultimately may promote well-being in cancer survivorship and have applications for addressing other health disparities. Including religious support and ethnic salience as important cultural factors in survivorship care plans may assist health professionals in promoting the well-being of African American and Latina BCS.
